# Commemorative Issue in Honor of Professor María Vallet Regí: 20 Years of Silica-Based Mesoporous Materials

**DOI:** 10.3390/pharmaceutics14010125

**Published:** 2022-01-05

**Authors:** Montserrat Colilla, Isabel Izquierdo-Barba, Gloria P. Rodríguez-Donoso, Natalia Otamendi-Vallet

**Affiliations:** 1 Departamento de Química en Ciencias Farmacéuticas, Instituto de Investigación Sanitaria, Universidad Complutense de Madrid, Hospital 12 de Octubre i+12, 28040 Madrid, Spain; 2 CIBER de Bioingeniería, Biomateriales y Nanomedicina, CIBER-BBN, 28040 Madrid, Spain; 3 E.T.S. Ingeniería Industrial, Institute of Energy Research and Industrial Applications, Universidad de Castilla-La Mancha (UCLM), 13071 Ciudad Real, Spain; Gloria.Rodriguez@uclm.es; 4 Agencia Española de Cooperación Internacional Para el Desarrollo (AECID), Oficina Técnica de Cooperación en El Salvador, Pasaje 2 Entre Calle La Reforma y Loma Linda, Colonia San Benito, 5707 San Salvador, Spain; nataota@gmail.com

This Special Issue entitled “Commemorative Issue in Honor of Professor María Vallet-Regí: 20 Years of Silica-Based Mesoporous Materials” arises from the initiative of the editorial team of *Pharmaceutics* to pay homage to Professor Maria Vallet-Regí for her ground-breaking pioneering scientific contribution to the field of silica-based mesoporous materials for biomedical applications.

Her exciting scientific career began in the 1970s in the Faculty of Chemistry at Universidad Complutense de Madrid (UCM), where she held an associated professor position, focusing her research activities in the field of solid-state chemistry and materials science (magnetic, conductive, catalytic, etc.). In 1990 she moved to the Faculty of Pharmacy at the same university as a full-time professor of inorganic chemistry. Then, she started a new stage focused on the field of *Biomaterials*, where she created, consolidated and still leads a new research group of recognized international prestige in the field of ceramic materials applied to biomedicine, nowadays known as the “Smart Biomaterials Research Group (GIBI)” [1,2]. 

Among the wide range of her scientific achievements, in this Special Issue we want to highlight her pioneering contribution in loading drugs into the pores of ordered mesoporous silica materials 20 years ago [3], which has motivated thousands of publications worldwide focused on the mesoporous materials for drug delivery and remains a scientific hot topic today (Figure 1).

María Vallet-Regí is a full-time professor at UCM and a group leader of the Biomedical Research Networking Centre in Bioengineering, Biomaterials and Nanomedicine (CIBER-BBN) and of the Research Institute of the Hospital 12 de Octubre (i+12). She is a chemist, researcher and lecturer. A recognized and highly cited researcher in biomedicine and nanoscience, she carries on with high dedication, vocation and passion in research activities in the fields of regenerative biomaterials and drug delivery systems. She has published 810 articles in peer-reviewed international journals, which have been cited more than 52,812 times and have an H-index of 108 [4].

Her scientific production also includes 12 patents, 300 plenary and invited lectures in national and international conferences, 20 books, 39 book chapters, 22 PhD Thesis and 10 Master’s thesis supervisions and more than 300 outreach activities.

Professor Vallet-Regí has received 21 awards, including many important international prizes, and she is member of the Spanish Academies of Pharmacy, Engineering, the Royal European Academy of Doctors, the International College of Fellows of Biomaterials Science and Engineering (FBSE) and the American Institute for Biomedical and Biological Engineering (AIMBE). She was a highly cited researcher in 2018 (Clarivate Analytics). This list recognizes world-class researchers selected for their exceptional research performance, demonstrated by the production of multiple highly cited papers that rank in the top 1% of citations per field and year in the Web of Science. A recent report released by Stanford University has included her among the 2% of the top researchers in the world. The database, which analyzes the career-long impact by researchers according to the Scopus database, was published in PLoS Biology in 2021 and ranked her as the eight most influential researcher worldwide in biomedical engineering and first female Spanish scientist to rank highest among her Spanish colleagues [5].

She was granted the title Doctor Honoris Causa by País Vasco University (2013), Jaume I University (2015) and Universidad de Murcia (2021). She has received the Jaime I Basic Research Award 2018, the Spanish National Research Award in Engineering 2008, the George Winter Award 2019, the Prix Franco-Espagnol 2000 from Societé Française de Chimie and the IUPAC 2013 Distinguished Woman in Chemistry or Chemical Engineering, among others. Very recently, she received the Medal of Merit for her 2019 research and university education as awarded by the Spanish government and recently received the Margarita Salas Prize 2021 awarded by the Community of Madrid.

She was included in the 1k Club of Chemistry of Materials and she received a European Research Council Advanced Grant (H2020, call 2015) aimed at designing a polyvalent mesoporous nanosystem to heal complex bone diseases, such as cancer, infection and osteoporosis.

The interest in silica-based mesoporous materials is due to their unique structural, textural and chemical surface properties, which make them suitable in a wide range of applications, from catalysis to biomedicine. This Special Issue is composed of 13 publications including research and review manuscripts related to the fundamental aspects of mesoporous materials (synthesis, characterization, functionalization, etc.) and potential applications, highlighting biomedicine.

From all over the world, outstanding contributions are included in this Special Issue. Comprehensive reviews describing the 20 years of advancements on mesoporous silica materials include the fundamental aspects to design and engineer drug delivery nanosystems for cancer and infection treatment, as well as mesoporous bioactive glasses and 3D scaffolds for bone tissue engineering. These revisions especially emphasize the contribution of Prof. María Vallet-Regí to this landscape through two pioneer research lines, drug delivery systems and bone tissue regeneration. Moreover, a relevant review focused on transmission electron microscopy and associated spectroscopic techniques revealed their pivotal role in the characterization of these structurally unique materials.

Advanced investigations to develop original mesoporous silica nanosystems with stimuli-responsive capability, including pH, ultrasounds and enzymes as release triggers, are also included. An in-deep study regarding the effect of ions released from mesoporous bioactive glasses on the different mechanisms involved in the bone regeneration process is also included. Finally, the incorporation of metal ions into mesoporous bioactive glass nanoparticles is proposed as an innovative approach to develop bone regeneration nanomaterials for multitherapy.

We are delighted to have been chosen by the journal as guest editors to organize this honorary issue, and we have counted on the best experts in this research field, both friends and scientists, to pay a deserved tribute to such a prominent scientific figure. Organizing this Special Issue and writing this editorial paper is very exciting and emotional for us, since we consider Marita, as all her friends affectionately call her, our “scientific mother”. We sincerely admire her open mind to change, her ability to adapt and stay at the forefront of cutting-edge science and technology from a multidisciplinary point of view. Thanks to all these skills, she has been able to transmit knowledge, enthusiasm, energy and passion for science to many young scientists (and not so young) and has become a role model for all of us.

In this editorial paper we have dared to invite two women who know certain facets of Marita that we would like to highlight to provide a more complete vision of the honoree (Figure 2).

On the one hand, we would like to highlight the commendable work in which Marita has been closely involved in recent years, which involves transmitting scientific knowledge to society through scientific outreach. To this aim, we have counted on the valuable help of Prof. Gloria Rodríguez-Donoso, coordinator of the Ingenious Women project G.R-D., who points out the following:

A vitally important facet to María Vallet-Regí, which is perhaps unknown to her international colleagues, is the incredible work she carries out in the field of scientific outreach, explaining her research not just to the scientific community, but to society at large. Marita is passionate about ensuring knowledge reaches all audiences, which is why it is not unusual to hear her speaking across a wide range of forums, especially national radio and television programs.

That said, Marita’s primary concern is explaining her work with biomaterials to young children. She has led her research group, GIBI, in preparing a range of videos that aim to bring the world of science to all kinds of people [1]. Just like the French series, “Once Upon a Time… Life”, they use formats which are designed to appeal to children. The episodes talk to us in simple, friendly language about bacteria and cells as “cabs” for drug-loaded nanoparticles for antitumoral therapy [6], as well as bioceramics for bone tissue regeneration and scaffolding [7], the design of biomaterials and surfaces with antibacterial properties [8] and many others [9,10,11,12,13].

Furthermore, Marita is very conscious of the low numbers of women enrolled in scientific-technological degree programs and the declining rates of women across the majority of STEM studies in general (science, technology, engineering and math). She is well aware of the importance of women being heavily involved and represented in these fields.

That is why, for many years, she has been involved in giving school talks to groups of boys and girls, not only in big cities but also in rural, sparsely populated areas. She tells them about her experiences, explaining why she decided to become a scientist and what her work involves, all in an easy-to-understand, friendly manner. This work has made Marita a true role model for Spanish teenagers and, in particular, young children. Thanks to her outreach work, many of them become interested in science and technology.

Marita has long supported “Mujeres Ingeniosas: la ingeniería en femenino” (Ingenious Women: female engineering), a project by the University of Castilla-La Mancha (Spain) which looks to involve more young girls in technology and engineering degrees [14]. A whole range of activities focused on breaking gender stereotypes are being carried out in order to achieve this goal, showing the importance of technology and highlighting the achievements of women who stand out in their fields, helping to make them social role models.

One of the main activities carried out in order to pursue this ideal is a traveling exhibition named “Mujeres Ingeniosas” (Ingenious Women), focusing on women who have excelled in the fields of engineering and technology. The exhibition chronicles the achievements of 17 women who have played key roles throughout history, all related to different aspects of engineering and technology. Alongside Ada Lovelace, Donna Strickland, Mildred Dresselhauss and Stephanie Kwolek, we find one panel dedicated to María Vallet-Regí. All of this ensures that her work is making an impact in a significant number of schools for many children, especially little girls who are deciding they want to be scientists when they grow up.

Finally, we want to underline her job as woman, mother and grandmother, and to this aim, we have had the pleasure of counting on the valuable collaboration of her daughter Natalia Otamendi-Vallet. In her own words, Natalia says:

Since I can remember she has been, is and will be a model of an independent, pragmatic, hard-working, committed, curious, restless, understanding and, of course, generous and loving woman. A mother who has taught my brothers and me that life is a fascinating gift to be enjoyed day by day, even if it sometimes presents us with seemingly insurmountable challenges. Marita, as she is known by her family and friends, teaches us that success has to be achieved day by day, with perseverance, effort, dedication and always as a team, where each member brings out their best qualities. In short, she is a brilliant and intelligent woman who inspires us, setting the bar very high for all of us who admire and love her, thus showing us the way forward. It is a source of pride to see how she is now reaping all the personal and professional rewards that she has cultivated and nurtured with love for so long.

She has always encouraged us to continue studying, thinking about the future, in terms of what we would like to do, and how we could apply what we are learning. She has managed, through her example, to ensure that her children are nowadays autonomous and independent, that we are working at what we like without feeling the hours go by and feeling useful to society, exactly as she does, although each one of us does it in a different sector and in a very different way.

Born in the Canary Islands, she has grown up and developed personally and professionally in Madrid, maintaining the Catalan customs of her parents, and enriching herself with the rest of the Spanish cultures with which she has been encountering.

She is a traveler woman, she loves to know different places and cultures, so her multiple knowledge and skills grow daily, without frontiers or barriers. She is able to apply something new to her life after each trip, and yet she remains humble and enjoying her routines that give her security and peace of mind. I remember at this moment with special affection our summer routine having aperitif in the best possible company with a red vermouth, olives and chips fried in Olive Oil Extra Virgin.

She gets inspired or escapes, as needed, by having a walk through Madrid’s streets or along a beach that comes within her reach. Although she doesn’t have a trace of a Canarian accent when she speaks, the ecosystem in which she was born has always been her weakness, and a little beach getaway is something she always enjoys and longs for.

Her work as a mother continues. We do not let her retire. During our childhood she taught us important values such as equality and solidarity. The three of us siblings collaborated equally in household chores or in deciding the destination of our next holiday. Of course, her vote was always of quality, but also fair and justified. We were also lucky enough to be able to ask her for help in understanding a chemical topic at school or at university, although, to be honest, this is something my friends enjoyed more than I did because, although we love each other deeply and understand each other very well, feelings can get in the way of teaching and bring out little moments of nerves or crises.

Yes, her work as a mother continues, caring about us every day, even though we live thousands of kilometers away. Always aware of her family’s achievements and joys, always concerned about our challenges and needs, always going ahead to give us a surprise and a joy, always there.

She is a technological and modern mother, always up to date, almost at the level of her teenagers grandchildren. She enjoys her grandchildren, probably more than she was able to do with her own children, without any worries or responsibilities beyond pampering them. Yes, “a techno and modern grandmother, a crack” say her grandchildren, and they don’t say “crack” because of her many awards and professional successes, but because she is simply the best in her day by day and in her personal life. For example, I would like to point out that she set up a little house in her garden for her grandchildren, not to play “little kitchens”, but to set up their own laboratory to make experiments that change the world, become perfumes or cure plants.

She is a super granny who also has her traditional and contradictory points that make her so special. Her kitchen is full of “modern gadgets” that she doesn’t use because there is nothing better than the oven or a pot simmering something. She also has millimeter laboratory measuring glasses that are only used by me when I go to her house, because she does everything “by eye”, with that fantastic intuition that characterizes her, and because by cooking that way everything tastes better every day, and they are never the same. Her exquisite culinary taste is also evident in the heritage she transmits to us. In this sense, it is a joy to hear any of her grandchildren, from their earliest childhood, ask her to cook a good red meat, some “chup-chup” cooked lentils or a delicious Spanish omelet.

She uses racks with test tubes as pencil holders on her desk and graduated test cylinders as vases for lilies, daisies and lavender. And it is that Marita has the scent of lavender, a scent that comforts, relaxes and stimulates at the same time, a magical scent that characterizes her, a scent of a woman, of a mother, of my mom”.

To summarize and conclude we would like to schematically illustrate Marita’s facets, which we have tried to introduce into this editorial paper (Figure 2).

Finally, just to say that it has been a great honor for us to edit this Special Issue to pay tribute to a worldwide-recognized scientist woman, our scientific referent and mentor, colleague and friend. *Nos sentimos privilegiadas de estar en tu equipo. Gracias, Marita.*

## Figures and Tables

**Figure 1:**
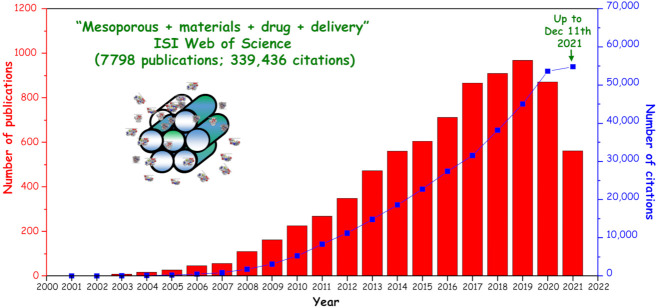
Number of publications per year indexed in the ISI Web of Science on the topic of “mesoporous” and “materials” and “drug” and “delivery” up to 11 December 2021.

**Figure 2:**
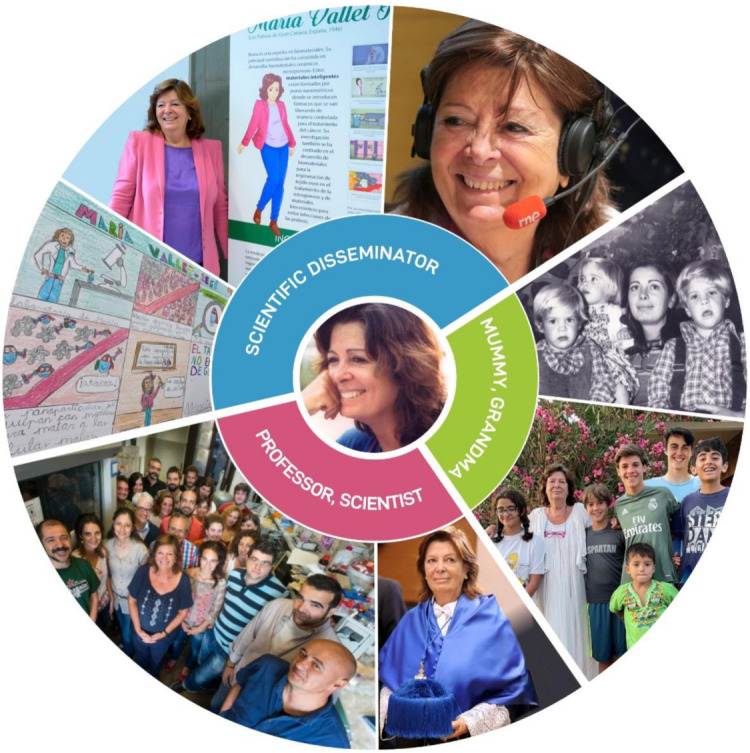
Schematical depiction of María Vallet-Regí’s different facets, which include professor and scientist, scientific disseminator, and mummy and grandma.
